# Kazakhstan Has an Unexpected Diversity of Medicinal Plants of the Genus *Acorus* (Acoraceae) and Could Be a Cradle of the Triploid Species *A. calamus*

**DOI:** 10.3390/plants13141978

**Published:** 2024-07-19

**Authors:** Dmitry D. Sokoloff, Galina V. Degtjareva, Carmen M. Valiejo-Roman, Elena E. Severova, Sophia Barinova, Victor V. Chepinoga, Igor V. Kuzmin, Alexander N. Sennikov, Alexander I. Shmakov, Mikhail V. Skaptsov, Sergey V. Smirnov, Margarita V. Remizowa

**Affiliations:** 1 School of Plant Sciences and Food Security, Faculty of Life Sciences, Tel Aviv University, Tel Aviv 6997801, Israel; 2 Biological Faculty, M.V. Lomonosov Moscow State University, Moscow 119234, Russia; degavi@mail.ru (G.V.D.); elena.severova@mail.ru (E.E.S.); margarita.remizowa@gmail.com (M.V.R.); 3 A.N. Belozersky Institute of Physico-Chemical Biology, M.V. Lomonosov Moscow State University, Moscow 119991, Russia; vallejo@genebee.msu.ru; 4 Faculty of Biology, Shenzhen MSU-BIT University, Shenzhen 518172, China; 5 Institute of Evolution, University of Haifa, Mount Carmel, Haifa 3498838, Israel; sbarinova@univ.haifa.ac.il; 6 Institute of Earth System Sciences, Leibniz University Hannover, 30167 Hannover, Germany; victor.chepinoga@gmail.com; 7 Faculty of Biology and Soil Sciences, Irkutsk State University, Irkutsk 664003, Russia; 8 Institute of Environmental and Agricultural Biology (X-BIO), Tyumen State University, Tyumen 625003, Russia; ivkuzmintgu@yandex.ru; 9 Botanical Museum, Finnish Museum of Natural History, University of Helsinki, 00100 Helsinki, Finland; alexander.sennikov@helsinki.fi; 10 South-Siberian Botanical Garden, Altai State University, Barnaul 656049, Russia; alex_shmakov@mail.ru (A.I.S.); serg_sm_@mail.ru (S.V.S.)

**Keywords:** Acorales, *Acorus verus*, anatomy, Asia, cryptic species, evolution, fertility, Irtysh River, molecular barcoding, polyploidy

## Abstract

The *Acorus calamus* group, or sweet flag, includes important medicinal plants and is classified into three species: *A. americanus* (diploid), *A. verus* (tetraploid), and *A. calamus* (sterile triploid of hybrid origin). Members of the group are famous as components of traditional Indian medicine, and early researchers suggested the origin of the sweet flag in tropical Asia. Subsequent research led to an idea of the origin of the triploid *A. calamus* in the Amur River basin in temperate Asia, because this was the only region where both diploids and tetraploids were known to co-occur and be capable of sexual reproduction. Contrary to this hypothesis, triploids are currently very rare in the Amur basin. Here, we provide the first evidence that all three species occur in Kazakhstan. The new records extend earlier data on the range of *A. verus* for c. 1800 km. Along the valley of the Irtysh River in Kazakhstan and the adjacent Omsk Oblast of Russia, *A. verus* is recorded in the south, *A. americanus* in the north, and *A. calamus* is common in between. We propose the Irtysh River valley as another candidate for a cradle of the triploid species *A. calamus*. It is possible that the range of at least one parent species (*A. americanus*) has contracted through competition with its triploid derivative species, for which the Irtysh River floods provide a tool for downstream range expansion. We refine our earlier data and show that the two parent species have non-overlapping ranges of variation in a quantitative metric of leaf aerenchyma structure.

## 1. Introduction

*Acorus* is the only genus of the family Acoraceae and the order Acorales, holding a phylogenetic position sister to the rest of the monocotyledons [1,2,3,4,5,6]. The genus comprises four to six species [7,8,9], all of high practical value, especially as medicinal plants [10,11,12,13,14,15,16,17,18,19,20,21]. The species of *Acorus* can be divided into two natural groups. The natural range of the *A. gramineus* group (1–3 species, apparently all diploid) is mostly restricted to tropical and subtropical regions of East and Southeast Asia [7,8]. The *A. calamus* group, as revealed by a recent taxonomic revision [9], includes three species with different ploidy levels: *A. americanus* (Raf.) Raf. (fertile diploid), *A. calamus* L. s.str. (sterile triploid), and *A. verus* (L.) Burm.f. (fertile tetraploid). *Acorus calamus* s.l. is famous for its diverse chemical compounds. For example, this species has been reported as the undisputed leader among Russian monocots in terms of the number of identified chemical compounds of an established structure (about 540) and the diversity of their biological activity [14]. Regrettably, almost all the enormous literature on chemical compounds does not consider the fact that what is currently called *A. calamus* in reality represents three distinct species.

According to our research [9], *Acorus americanus* is known to occur in North America (Southern Canada and the northern United States) and in northern Asia, where it is widely distributed in Russian Siberia (reaching central Yakutia in the north, Omsk Oblast in the west, and Kamchatka in the east) and extending southward into Mongolia and China (at least Inner Mongolia). The Asian and American accessions of *A. americanus* are classified as two subspecies (subsp. *triqueter* and subsp. *americanus*, respectively). Wherever it occurs, *A. americanus* mostly sets fruits with seeds and is considered to be a self-compatible species. Unless pre-historic human impact is involved, *A. americanus* is likely native in most parts of its range. *Acorus verus* is known to occur and set fruits in the southern part of the Russian Far East, Japan, Korea, and China (central, eastern, north-eastern, and northern regions to the east of 109 °N). In addition, *A. verus* occurs in tropical South and Southeast Asia, where it sets no seeds because of self-incompatibility and/or the lack of specialised pollinating beetles and propagates vegetatively. It is likely that *A. verus* was introduced here by humans as a medicinal and ritual plant [9]. *Acorus calamus* evolved through hybridisation between *A. americanus* and *A. verus*. It propagates only vegetatively. A single origin for *A. calamus* has been hypothesised because it consistently possesses the same plastid haplotype as the diploid *A. americanus* [9]. *Acorus calamus* occurs in Europe and the United States (extending to the extreme southwest of Canada), where it is introduced by humans and naturalised [11,22,23,24,25,26,27,28]. The triploid *Acorus* is also widely cultivated and grows spontaneously in various regions of Asia, including India, where it was thought by some authors to first evolve (e.g., [29,30,31]). The origin of the triploid *A. calamus* in tropical Asia is, however, problematic because at the current state of knowledge, there is no region within South Asia where the two parental species (*A. americanus* and *A. verus*) grow together, and both exhibit sexual reproduction. Before the present study, the basin of the Amur River in the Far East of Russia and adjacent regions of China was the only plausible candidate area for the origin of the triploid *A. calamus* [9,25]. 

Here, we provide an update on the taxonomic diversity of *Acorus* in Kazakhstan and adjacent parts of Western Siberia. We had only limited collections from Kazakhstan while preparing our taxonomic account for temperate Asia. All the samples we had from Kazakhstan belonged to the sterile triploid species *A. calamus* [9]. This was in agreement with the idea that Central Asia has a restricted occurrence and low diversity of *Acorus*. For example, the classical monograph ‘Flora of Kazakhstan’ [32] accepted one species (*A. calamus* s.l.) and reported that the plant has its origin in India, the seeds do not ripen, and the reproduction is exclusively vegetative. With more collections studied, here, we document the occurrence of all three species of the *A. calamus* group in Kazakhstan. Newly discovered localities in Kazakhstan unexpectedly broaden the range of *A. verus*. They are located more than 1800 km from the closest known occurrences of the tetraploid *Acorus* in northern India and more than 2200 km from the closest record of *A. verus* in China (prov. Shaanxi) [9]. We highlight the importance of the Irtysh River valley for understanding the historical biogeography of *Acorus* and show that along with the Amur River basin it could be a likely candidate for a cradle of the triploid species *A. calamus*. Keeping in mind the importance of this river system, we extended our interest from Kazakhstan to the adjacent Russian part of the Irtysh basin in Omsk Oblast.

Our earlier study, using extensive data from across Asia, Europe, and North America, revealed that *A. calamus*, *A. americanus*, and *A. verus* can be precisely distinguished using sequences of the nuclear ribosomal internal transcribed spacer [9]. *Acorus verus* differs from *A. americanus* in as many as 12 single nucleotide substitutions plus 10 indels in nuclear ribosomal ITS sequences (nrITS). We called the sequence found in *A. americanus* ribotype I and that of *A. verus* ribotype II. We found no infraspecific variation in our extensive material from Russia, Mongolia, India, and the USA. *Acorus calamus* has both ribotypes with no traces of contamination, apparently because of its exclusively clonal reproduction. Therefore, nrITS is an efficient tool for molecular barcoding *Acorus* species [9]. DNA content as inferred from flow cytometry provides another direct tool to distinguish between the triploid *A. calamus* (2C = 1.23–1.31 pg)*,* the diploid *A. americanus* (2C = 0.88–0.95 pg), and the tetraploid *A. verus* (2C = 1.47–1.61 pg) [9,33,34]. Seeds of *A. americanus* (morphotype I) and *A. verus* (morphotype II) differ in the absence vs. presence of long, branched hairs in the micropylar region [9]. *Acorus calamus* never produces any seeds, and its pollen shows a high percentage of sterility, as measured through its stainability with acetocarmine or cotton blue in lactophenol [9,11,26,35]. The pollen of two other species is largely fertile (the stainability is, in most cases, over 90%). Our earlier study covered leaf anatomy in 449 individual plants of *Acorus* from 253 localities in Austria, Belarus, Bhutan, Canada, China, Germany, India, Japan, Kazakhstan, Mongolia, Nepal, Russia, Ukraine, the USA, and Uzbekistan [9]. We found that the mean cell number in the septa of leaf lamina aerenchyma as seen in cross-section (*N*_cell_) ranges from 0.5 to 3.2 (1.02–2.85 in 95% of samples studied) in *A. verus* and from 2.95 to 7.8 (3.40–7.15 in 95% of samples studied) in *A. americanus*. The range of the leaf aerenchyma metric in *A. calamus* (*N*_cell_) overlapped with those of the two parent species. To summarise, various tools for identification showed excellent correlations with each other. Each of the two tools—flow cytometry and nrITS barcoding—alone is sufficient for precise identification. Seed micromorphology, leaf anatomy, and pollen stainability, taken together, also allow for precise identification. When seeds are available or when the pollen is sterile, seed and pollen characters alone provide for precise identification. On the other hand, features of external morphology (e.g., those available in scanned herbarium specimens) are insufficient for the precise recognition of the three species, at least in Asia. They can therefore be considered cryptic species in the wide sense of the term [36], or pseudocryptic, because of the existence of micromorphological differences [37].

## 2. Materials and Methods

We followed all protocols used in our earlier study [9], including those of DNA extraction, amplification, sequencing, and alignment, flow cytometry, scanning electron microscopy (SEM), anatomy with light microscopy (LM), pollen stainability, and creating a distribution map. Herbarium collections of AA, ALTB, LE, MW, and TK were used (Table 1). Identification tools applied to each examined specimen varied depending on the age of the specimen, its developmental stage, and the parts kindly allocated for destructive sampling by the curators. GenBank accessions for sequences of nrITS are provided in Table 2.

## 3. Results

### 3.1. Taxonomic Identification of Herbarium Collections

**Figure 1:**
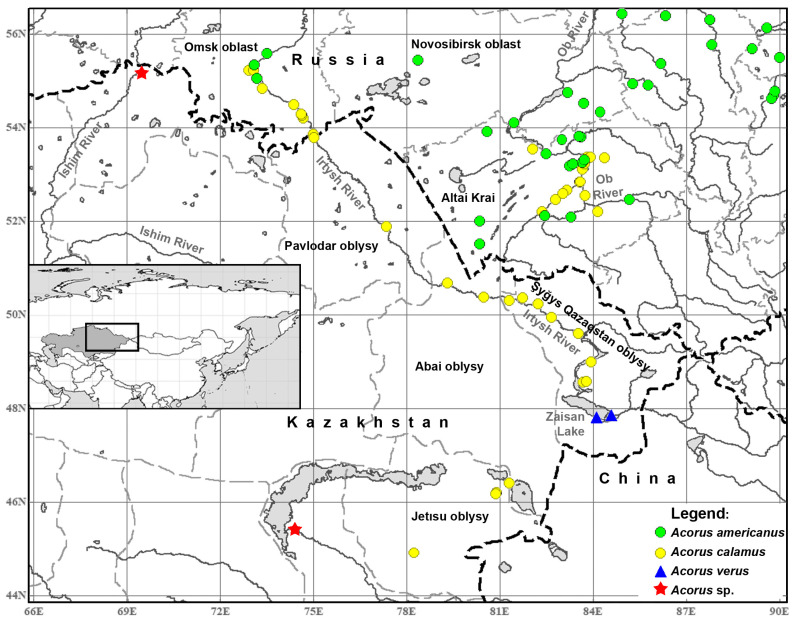
Distribution of *Acorus* species in Kazakhstan and adjacent parts of Russia. Inset: Kazakhstan in the map of Asia and the position of the main map. Red stars indicate records of *Acorus* from Zhideli channel in the valley of the Ili River [38] and from the Ishim River in Soltüstık Qazaqstan oblysy [39] for which we had no access to voucher specimens. The only specimen of *A. americanus* from Pavlodar oblysy has no precise locality information and is therefore omitted from the map.

Our data document the occurrence of all three species of the *A. calamus* group in Kazakhstan and show that both *A. calamus* s.str. and *A. americanus* occur in Omsk Oblast of Russia. Full lists of species records in each region based on the present study are provided in Table 1. Table 2 summarises diagnostic characters used to identify each specimen. Figure 1 provides a distribution map of *Acorus* species in Kazakhstan and adjacent parts of Russia. The map shows only that part of Kazakhstan where *Acorus* does occur according to examined collections.

The present work revealed the same two haplotypes of nrITS as the earlier study, and the occurrence of haplotype I, II or their combination allowed identification of *A. americanus*, *A. verus*, and *A. calamus*, respectively (Table 2). Figures of the nuclear DNA content of 2C = 1.24–1.26 pg and/or very low percentages of stainable pollen grains allowed for the precise recognition of the sterile triploid *A. calamus*, while the absence of any seed set in well-postanthetic inflorescences was considered indirect evidence of sterility (Table 2). The main metric of leaf anatomy used here was *N*_cell_, which is the mean cell number per septum in leaf lamina aerenchyma, as seen in a cross-section. Like in the previous study, the mean was calculated using cell counts in at least 20 septa in each specimen. As such, *N*_cell_ does not provide a useful tool to identify triploids, but the range of variation of *N*_cell_ found here in examined samples of *A. calamus* (2.15–3.60) fits very well with the range observed for this species in other regions of Eurasia [9].

**Figure 2:**
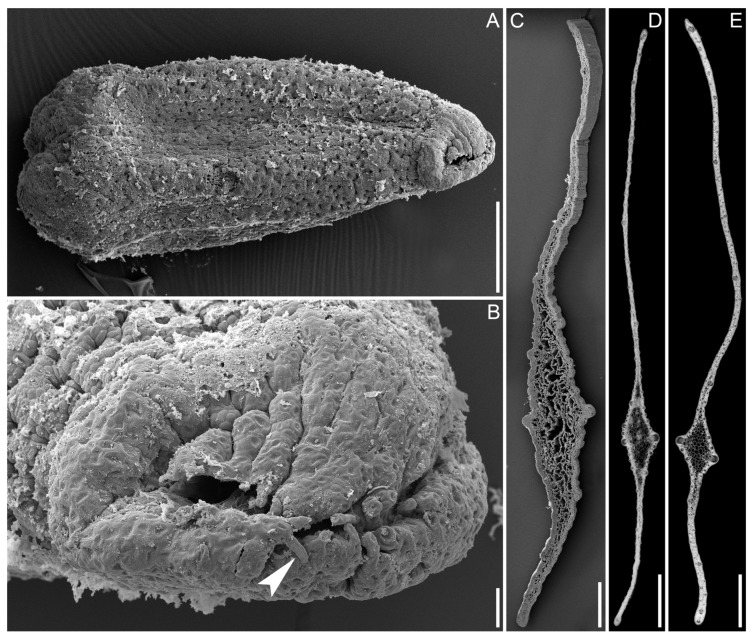
Micromorphology of specimens of *Acorus americanus* and *A. verus* from Kazakhstan. (**A**–**C**), *Acorus americanus* (*Medvedev & Melvil s.n.*, AA002771). (**A**), entire seed, micropylar side right. (**B**), frontal view of the micropylar side; arrowhead, very short unbranched hair. (**C**), leaf in cross-section, abaxial side bottom. (**D**,**E**), *Acorus verus*, leaves in cross-section, abaxial side bottom. (**B**), *Pechnikova s.n.*, (AA002779). (**C**), *Samusev s.n.* (AA002770). (**A**–**C**), SEM; (**D**,**E**), LM. Scale bars = 0.5 mm (**A**,**C**), 50 µm (**B**), 1 mm (**D**,**E**).

**Figure 3:**
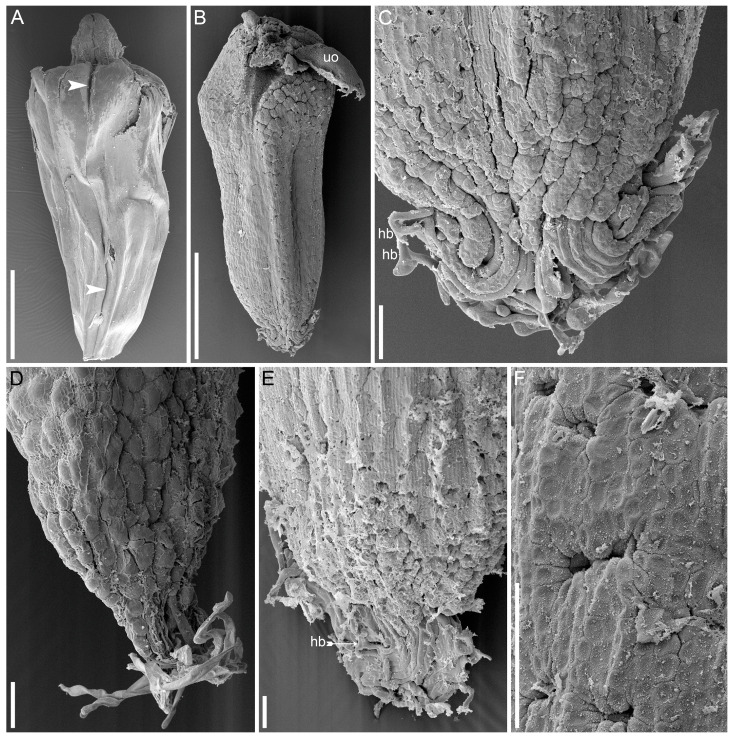
Fruit and seed micromorphology of specimens of *Acorus verus* from Kazakhstan (SEM). (**A**), mature fruit, stigmatic side up. (**B**), seed, micropylar side down. (**C**–**E**), seeds, details of the micropylar side with long hairs. (**F**), detail of the seed coat surface; depressions indicate the positions of the presumed ethereal oil cells. (**A**,**E**,**F**) *Pechnikova s.n.* (AA002780). (**B**,**C**), *Pechnikova s.n.* (AA002779). (**D**), *Samusev s.n.* (AA002770). Arrowhead, border between adjacent carpels; hb, hair branching; uo, unfertilised ovule. Scale bars = 1 mm (**A**,**B**), 0.1 mm (**C**–**F**).

**Figure 4:**
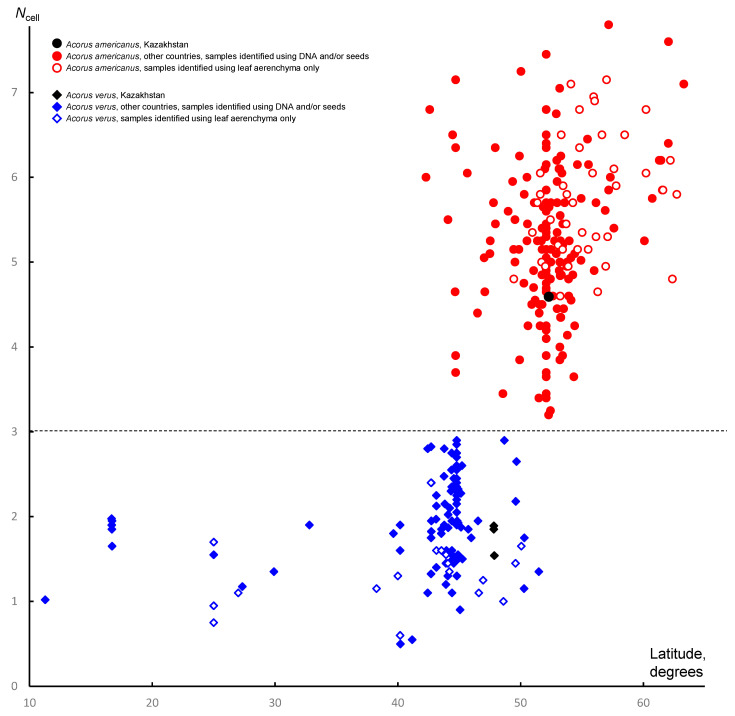
Quantitative data on the variation of leaf aerenchyma in the fertile species *Acorus americanus* and *A. verus* as related to the geographical latitude of collection localities. *N*_cell_ is the mean number of cells in the septa between the air canals visible in the cross-section of the leaf blade. The mean was calculated using cell counts in at least 20 septa in each specimen. The latitude of the Kazakhstan specimen of *A. americanus* is arbitrary indicated as that of Pavlodar city. Data on countries other than Kazakhstan are taken from [9]. See text for discussion on samples *Golde s.n.* (LE01249647) and *Bobrov & Mochalova 694* (MW0163317). The dashed line shows that *N*_cell_ = 3 serves as a clear boundary between the ranges of variation recorded in the two fertile species.

Seed micromorphology provided unequivocal evidence for the identification of collections of *A. americanus* and *A. verus* from Kazakhstan. The specimen of *A. americanus* (Figure 2A,B) has well-developed seeds bearing only rare, very short (<0.05 mm), unbranched hairs (seed morphotype I [9]). Specimens of *A. verus* (Figure 3) also have fruits with well-developed seeds, but these bear numerous long (>0.2 mm), branching hairs (seed morphotype II [9]). Figures of *N*_cell_ inferred from the specimens of *A. verus* and *A. americanus* examined here (Table 2) fit very well with the ranges of variation inferred for each of these species in our earlier study (Figure 4); also, these figures do not fall into the interval of *N*_cell_ = 1.95–4.15 that includes 95% of samples of *A. calamus* included in the earlier study [9].

**Figure 5:**
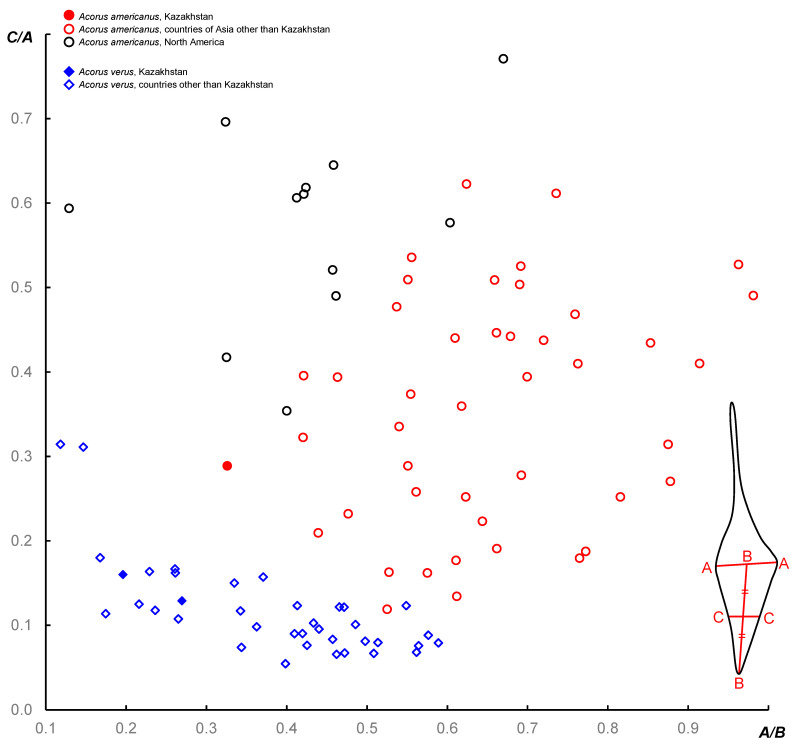
Quantitative analysis of the shape of lamina cross-section in the fertile species *Acorus americanus* and *A. verus*. Inset shows a diagram of leaf lamina in cross-section with a legend to the metrics A, B, and C. Data from localities other than in Kazakhstan are taken from [9].

We recognised two subspecies of *A. americanus* based on characters of leaf anatomy and morphology [9]. The American taxon, subsp. *americanus*, has leaves typically with several equally developed large vascular bundles on either side. Leaves of the Asian subsp. *triqueter* in most instances have a conspicuous major bundle. In addition, specimens from Asia and North America tend to differ in the shape of the leaf lamina, as seen in a cross-section. The Kazakhstan specimen of *A. americanus* studied here has the quantitative metric of the shape of the lamina cross-section that does not allow its unequivocal placement into either subspecies (Figure 5). Leaf vasculature is also variable in this specimen, with the occurrence of one, two, or three larger veins, sometimes in various parts of the same leaf.

The material of *A. verus* from Kazakhstan is similar to collections of this species from other countries regarding the quantitative metric of the shape of the lamina cross-section.

### 3.2. Notes on Critical Historical Specimens

*Medvedev & Melvil s.n.* (AA002771) is the only specimen of *A. americanus* known so far from Kazakhstan. It was collected in Pavlodar Krai (now Pavlodar oblysy) in 1930 but has no precise locality information. We believe that it indeed comes from Kazakhstan, because the collectors were employed by the Kazakhstan Institute for Soil Studies (operated in Kyzyl-Orda Town during 1929–1933) for surveying pastures and hayfields [40] (Bykov & Kubanskaya, 1960), and, according to further herbarium specimens kept at TK, it is known that they conducted a vegetation survey of Pavlodar Krai during this year. Furthermore, the occurrence of *A. americanus* in areas of Kazakhstan adjacent to the border with Altai Krai of Russia looks more than plausible. In Altai Krai, the species was found in wetlands associated with so-called ribbon pine forests [9]. The ribbon forests are long and narrow belts that extend for hundreds of kilometres from the Ob River valley in Russia in the east towards the Irtysh River valley in Kazakhstan in the west.

*Karelin & Kiriloff 1066* (MW0812267, TK) has no label information on the collection locality; only the year is indicated (1840). G.S. Karelin and I.P. Kirilov were famous and pioneering plant collectors in Central Asia, and the history of their collections is known in detail [41]. A full catalogue of their collections made in 1840 is published [42]. While more than one locality is indicated for many other species in the catalogue, only one is provided for *Acorus* (“In paludosis prope Buchtarminsk. Fl. Julio.”), which indicates the precise geographical origin of the collection (Bukhtarma, Bukhtarminsk or Ust-Bukhtarminskaya, formerly populated place at the confluence of the Bukhtarma and Irtysh rivers in Kazakhstan).

*Golde s.n.* (LE01249648) and *Golde s.n.* (LE01249647) are specimens with similar but not completely identical labels. Karl L. Golde made extensive plant collections from the present-day Omsk Oblast of Russia during his service as pharmacy administrator in Omsk for the Ministry of War of the Russian Empire in 1884–1886 [43]. During that period, his collecting activities and botanical works were facilitated and financed by the West-Siberian Department of the Imperial Russian Geographical Society [44]. Golde accurately labelled his herbarium specimens with locality information and collection dates according to the Julian calendar (as evidenced by explanations of his collection activities [45]. The Siberian collections of Golde have been acquired by predecessors of the present-day Komarov Botanical Institute (LE) in three ways. One part was sent by the collector for identification and revision to K.F. Meinshausen, who was a conservator at the Botanical Museum of the Imperial Saint Petersburg Academy of Sciences [46]. Such specimens (LE01249647) were curatorially labelled by Meinshausen. A smaller portion of this collection was deposited by Golde in 1894 to the Imperial Botanical Garden [47]. Finally, the main set of Golde’s collections was purchased by the Botanical Museum after his death [48]. The latter specimens (LE01249648) bear original collector’s labels, and all specimens (acquired in 1886 and 1907) were curatorially labelled by Litvinov with printed forms of the Botanical Museum (Sennikov, pers. obs. in herbarium collections). The Museum’s collections were kept unmounted at that time, and specimens of the same species may have been accidentally mixed during that period, so the geographical origin of some particular specimens may become unreliable (Sennikov, pers. obs. in herbarium collections).

We confidently identify LE01249648 as *A. calamus*. This specimen has an original handwritten label of Golde, but the plant material includes only a spadix with its peduncle. Neither vegetative leaves nor a spatha are available. LE01249647 has two plant fragments that are not connected to each other. The left fragment is an inflorescence that we with certainty identify as *A. americanus*. The right fragment is vegetative. In our previous study [9], we used LE01249647 as a representative specimen of *A. americanus* from Omsk Oblast, and its species identification was based on the left fragment bearing seeds. We inferred data on leaf anatomy from its vegetative fragment and interpreted the results as belonging to *A. americanus*. The value of *N*_cell_ = 2.95 that we found in *Golde s.n.* (LE01249647) was the lowest among the total of 173 samples of *A. americanus* collected from all across the species range, including Western and Eastern Siberia, Mongolia, the Far East of Russia, and North America [9]. Now, we can take into consideration the specimen *Golde s.n.* (LE01249648) that has a similar label and belongs to *A. calamus*. Since the specimens had been originally kept loose together with other collections, we see no proof that the right fragment of LE01249647 belongs to *A. americanus*. It could well belong to *A. calamus* and be part of the collection mounted in LE01249648. The figure of *N*_cell_ = 2.95 falls among typical values of *A. calamus.*

It is a good question whether Golde’s collections indicate that *A. calamus* and *A. americanus* were growing side-by-side near Omsk in the 1880s. Based on our extensive fieldwork experience, we are not aware of other localities where these two clonal species grow in mixed populations. A report of diploids and triploids growing together in Iran [49] requires further study; in the absence of precise taxonomic identification, we consider the chromosome counts in that work unreliable because the characters other than ploidy level were not investigated. LE01249648 has a handwritten collector’s label with a more precise locality, ‘behind the camp’, which should be read as behind the military camp. Where the military camp was located at that time near Omsk is well documented. One of us (I.V.K.) visited the locality in 2023 and found that *Acorus* no longer occurs there because an embankment of the Irtysh River has been constructed since Golde’s time. Both LE01249648 and LE01249647 indicate the collection date as 28 July 1886 according to the Julian calendar, which corresponds to 9 August in the Gregorian calendar. The inflorescence of *A. calamus* in LE01249648 is at the male stage of anthesis, which would be not realistic for a plant collected in August. This stage of *Acorus* anthesis normally takes place in June and early July in the southern part of Siberia. For example, specimens of *A. calamus* collected 20 km northeast of Omsk on 31 July 1900 (Julian calendar) are postanthetic (*Skalozubov 1384,* LE01249642, LE01249645). The inflorescence of *Golde s.n.* (LE01249647) has fruits with well-developed seeds. This stage perfectly fits the first decade of August of the modern calendar. We conclude that the specimen of *A. americanus* was collected by Golde in Omsk, whereas the material of *A. calamus* in LE01249648 and LE01249647 comes from another collection of unknown origin. Thus, the collections of Golde should not be taken as proof that diploid and triploid plants of *Acorus* occurred in a mixed population.

## 4. Discussion

### 4.1. Acorus verus and A. americanus Have Non-Overlapping Ranges of Variation in Quantitative Leaf Anatomy

Röst [25] compared leaf aerenchyma structure in *Acorus* to ploidy level in a common garden experiment. It was logical to expect that with the use of herbarium collections covering entire geographical ranges of the diploid *A. americanus* and the tetraploid *A. verus*, the picture of quantitative differences between diploids and tetraploids would become less sharp. Climatic conditions vary considerably across species ranges in *Acorus* (for example, between middle Yakutia, Altai Krai, and Minnesota in *A. americanus*). Moreover, when public herbarium collections are used, one should consider that they were made in different seasons and that no more than one leaf per specimen can be normally investigated. Some herbarium specimens allowed for sampling vegetative leaves, while for others, only the leaf was associated with inflorescence (the spatha). Nevertheless, using a refined metric of leaf aerenchyma (*N*_cell_), our earlier study found only a narrow zone of overlapping in variation ranges between 173 wild-source samples of *A. americanus* and 132 samples of *A. verus* [9]. In the present study, we were able to reconsider the interpretation of leaf anatomy in *Golde s.n.* (LE01249647) that had the lowest figure of *N*_cell_ (2.95) in our data set of *A. americanus*. Our new data show that this count cannot be safely attributed to *A. americanus* and most likely belongs to *A. calamus*. With the count of 2.95 omitted, the ranges of variation of *N*_cell_ no longer overlap between the diploid *A. americanus* and the tetraploid *A. verus*. Having this conclusion, we decided to re-investigate our earlier data on the highest figure of *N*_cell_ in *A. verus*, namely *N*_cell_ = 3.18 for *Bobrov & Mochalova 694* (MW0163317, Khabarovsk Krai, Russia). This specimen has only one vegetative leaf. We re-sampled and re-investigated anatomically the *Bobrov & Mochalova 694* specimen. For a better confidence, we studied two different sections of this leaf and in each instance counted cells in 50 aerenchyma septa. These two sections provided *N*_cell_ values of 2.22 and 2.18, which are rather typical of *A. verus*. We therefore conclude that a lab mistake likely took place in our earlier study of *Bobrov & Mochalova 694.*

With the refined dataset, *A. americanus* and *A. verus* have completely non-overlapping ranges of the variation of leaf aerenchyma. The difference can be easily described in an identification key: *A. verus* has *N*_cell_ < 3 while *A. americanus* has *N*_cell_ > 3 (Figure 4).

### 4.2. The Importance of the Discovery of A. verus in the Lake Zaisan Area

This is the first report of A. verus in Kazakhstan and Central Asia. The importance of our new records from the Lake Zaisan area is not limited by the fact that they are at least 1800 km away from any previously documented locality of the species. All earlier records of *A. verus* were located within the Asian monsoon precipitation domain (as defined by Wang and Ding [50]) or close to its borders. The conditions in eastern Kazakhstan are far from the monsoon climate. Earlier records of *A. verus* lie within the area with August mean precipitation above 2.88 mm/day (and mostly with even much higher precipitation in August). In contrast, August mean precipitation is below 0.93 mm/day in the Lake Zaisan area [51]. Therefore, our new data considerably broaden our understanding of the climatic requirements of *A. verus.* They further support the idea that temperature regime plays a more important role than precipitation in shaping distribution ranges of aquatic species [9,52]. With respect to temperature regime, our new records fit well the earlier idea that *A. verus* requires a warmer climate than *A. americanus,* and therefore the two species tend to separate geographically along a latitudinal gradient (Figure 1 and Figure 4).

All three samples of *A. verus* from Kazakhstan have fruits with well-developed seeds. This is in contrast with the material of this species from tropical Asia, including India, where *A. verus* never sets seeds despite high pollen fertility. We speculated that the absence of the seed set in tropics is because of self-incompatibility within large clones derived from ancient introduction and/or because of the absence of pollinators. Both *A. verus* and *A. americanus* are specialised for pollination by two beetles, *Platamartus jakowlewi* and *Sibirhelus corpulentus* (Kateretidae), that lay eggs into the inflorescences [9,53,54]. All known records of these beetles do not extend beyond the area where *Acorus amerianus* and/or *A. verus* set fruits. *Sibirhelus corpulentus* is known from Russia (Altai Krai, Irkutsk Oblast, Primorsky Krai) and Japan; *P. jakowlewi* is recorded from Altai Krai, Republic of Tuva, Yakutia, Irkutsk Oblast, and Krasnoyarsk Krai in Russia as well as from Japan [9,53,55]. Now, we should expect findings of *Platamartus jakowlewi* and *Sibirhelus corpulentus* in Kazakhstan. These records will indicate a re-consideration of the climatic requirements of the beetles, because no species of *Acorus* was previously known to set fruits in a climate with as dry summer weather as in the Lake Zaisan area.

It is interesting that one of the Kazakhstan specimens of *A. verus* (*Samusev s.n.*, AA002770) has been examined by K.V. Dobrokhotova, which is evidenced by her identification as *A. calamus* s.l. The whole label, including the name of the expert, is written by the same hand, most likely soon after the sample was collected in 1950. The fact that Dobrokhotova [32] indicated the absence of seed ripening in *Acorus* in Kazakhstan shows that she did not pay attention to the occurrence of fully ripening and abscising fruits with mature seeds in this specimen. It is most likely that the information on non-ripening seeds was taken from the general account of *Acorus* for Flora of the USSR [56], where this feature is mentioned in the context of the introduction of *A. calamus* in Europe, but the text can be read as if the author intended to extend the absence of seed ripening into the Asiatic part of the range, too.

The fact that *A. verus* sets seeds in Kazakhstan suggests that it is native rather than introduced here. The habitats of *A. verus* in the Lake Zaisan area, as indicated in herbarium labels, suggest quite natural wetlands. All three collections of *A. verus* were made before the construction of the Bukhtarma hydroelectric power station that started operating in 1960s and changed the ecosystem of Lake Zaisan by elevating its water level by several meters. Therefore, the localities of *A. verus* discussed here are currently underwater. New fieldwork is needed to figure out whether the species still occurs in Kazakhstan. We believe that it does, because large wetlands still occur in the delta of the Black Irtysh [57,58,59], apparently close to the locality of *Samusev s.n.* (AA002770). The collector indicated the locality as ‘Old Irtysh’, which is one of the channels of the delta of the Black Irtysh [58]. Dimeyeva et al. [59] mentioned ‘*Acorus palustris*’ while characterising the vegetation of the wetlands of the Black Irtysh delta.

### 4.3. Taxonomy Is a Key Tool in All Studies Related to Medicinal Plants

Our new data show that Kazakhstan has important genetic resources of *Acorus*, and these resources can be of practical use. *Acorus* is well-known as a medicinal plant. Studies based on *Acorus* collections from other countries revealed important correlations between the ploidy level and the composition of essential oils, especially the content of β-asarone, which is highest in tetraploids [29,59,60,61,62]. Unfortunately, published studies of chemical composition, medicinal properties of *Acorus* and stocks of these medical plants in Kazakhstan [39,63,64,65,66,67,68] did not consider the ploidy level of the material. At the current level of knowledge, as outlined above, plants with different ploidy levels can be easily distinguished using micromorphology because they belong to different taxonomic species. A phytochemical investigation of *A. verus* from the Lake Zaisan area and an analysis of its resources will be important.

We highlight the critical importance of botanical taxonomy in all studies related to medicinal plants. Not only is it important with respect to plant resources existing today, but also with respect to the history of the uses of spices and medicinal plants. It is still unclear whether the calamus of the Bible should be identified as *Acorus* [10]. It is equally unclear whether *Acorus* or *Cymbopogon* was used as an important aromatic plant in ancient Mesopotamia [69]. Reports that certain Ancient Greek and Ancient Egyptian perfume or medicinal recipes included exactly *Acorus calamus* var. *angustatus* [70] seem not to be supported by convincing evidence. *Acorus calamus* var. *angustatus* was suggested by Röst [25] as a name for tetraploid plants, but this name is illegitimate and cannot be used for any taxon [34]. More importantly, with only verbal descriptions in the historical literature, there is no chance of distinguishing the tetraploid *Acorus* (*A. verus*) from diploid (*A. americanus*) and triploid (*A. calamus*) plants. However, if the archaeological record has preserved rhizomes, we believe that paleogenetics may help in disentangling their taxonomic identity. Such research will be of mutual interest to botany and history.

### 4.4. Kazakhstan Could Be a Cradle of the Triploid Species A. calamus

Some early researchers suggested the origin of *A. calamus* in tropical Asia (e.g., [71,72]). They accepted the wide circumscription of the species to include both fertile and sterile plants. A more specific idea of the origin of the triploid *A. calamus* in the Himalayan region of India has been proposed [29,30,31]. The absence of the fruit set of both sterile and fertile plants in tropical Asia makes this hypothesis problematic [9]. Subsequent research led to an idea of the origin of the triploid *A. calamus* in the Amur River basin in temperate Asia because this was the only region where both diploids and tetraploids were known to co-occur and be capable of sexual reproduction [9,25]. However, triploids are currently very rare in the Amur basin. For example, even though rare diploids are recorded along with the common tetraploids near Lake Khanka in Primorsky Krai, our study of voucher specimens allowed for rejecting an earlier report of triploids from this area [73], and no triploid plant was found during our extensive fieldwork there [9].

Here, we provide the first evidence that all three species of the group occur in Kazakhstan. Along the valley of the major River Irtysh in Kazakhstan and the adjacent Omsk Oblast of Russia, *A. verus* is recorded in the south, *A. americanus* in the north, and *A. calamus* is common in between. We propose the Irtysh River valley as another candidate for a cradle of the triploid species *A. calamus*. It is possible that the range of at least one parent species (*A. americanus*) has contracted through competition with the triploid species, for which the Irtysh River floods provide a tool for downstream range expansion. The Irtysh River valley has been at an intersection of trade routes and human migrations since pre-historic times. One can easily imagine human-mediated dispersals of triploid plants from the Irtysh River valley to the Ob River valley in Altai Krai, where the species is currently common, southwards to other regions of Middle Asia and eastwards to China. Molecular phylogeographic analysis of the entire group will help in testing the Amur basin and the Irtysh valley hypotheses of the origin of *A. calamus*. The distance between the two potential regions of the origin of *A. calamus* exceeds 3000 km. We hope that the two parent species exhibit a certain geographical differentiation at the molecular level over such distances and that this differentiation will be sufficient for the evaluation of the cradle of *A. calamus.*

The Irtysh and Ob valleys provide model systems to investigate the relative impacts of ice-mediated downstream dispersal, differential climatic requirements and potential competition between different species of *Acorus*. The Irtysh River valley has three species of *Acorus*, whereas the Ob River valley has only two species (*A. calamus* and *A. americanus*). In both instances, species differ in their distribution along the valley, with at least *A. calamus* being common in the areas where it occurs. *Acorus* plants (at least those of *A. calamus*) are perfectly adapted for vegetative dispersal along large river valleys where their rhizomes can be transported during spring floods as frozen into ice [39,74]. In both Irtysh and Ob valleys, the direction of the downstream transport is towards the north, and the plants thus potentially migrate into areas with a less optimal temperature regime. It has been hypothesised that the extensive vegetative propagation of the triploid *A. calamus* provides certain adaptive advantages over the diploid *A. americanus* in floodplains of a large river [33]. This idea can be tested by a targeted study of all populations of *Acorus* at the zone of overlap between two species. Potential zones of overlap between *A. calamus* and *A. americanus* in Novosibirsk Oblast of Russia and between *A. verus* and *A. calamus* in Şyğys Qazaqstan oblysy of Kazakhstan are affected by the construction of hydroelectric power stations. On the other hand, the contact zone between diploids and triploids in Omsk Oblast is not affected and would serve an excellent area for ecological research.

## 5. Conclusions

1. All three species of the *Acorus calamus* group occur in Kazakhstan.
2. Along the valley of the Irtysh River in Kazakhstan and the adjacent Omsk Oblast of Russia, the tetraploid *A. verus* is recorded in the south, the diploid *A. americanus* in the north, and the sterile triploid *A. calamus* is common in between.
3. The Irtysh River valley could be a more plausible area of the origin of the triploid species *A. calamus* than the Amur River basin. The dilemma will be disentangled using molecular phylogeography.
4. The Irtysh valley provides a model system to investigate relative impacts of ice-mediated downstream dispersal, differential climatic requirements and potential competition between different species of *Acorus*.
5. Micromorphology allows for precise diagnostics of all three species of the *A. calamus* group, except for vegetative specimens. The two fertile species have non-overlapping ranges of variation of leaf aerenchyma.
6. The discovery of *A. verus* in the Lake Zaisan area suggests the overlooked occurrence of its specialised beetle pollinators *Platamartus* and *Sibirhelus* in the region and facilitates further work on the genetic resources of the important medicinal plant in Kazakhstan.

## Figures and Tables

**Table 1:** Examined specimens of *Acorus* from Kazakhstan and Omsk Oblast of Russia and their taxonomic identities as inferred from our data.

*Acorus americanus* (Raf.) Raf.
**RUSSIA. Omsk Oblast:** [Sargatskiy Rayon,] river Zamarayka, *V. Drovorub s.n.* (LE01249644); [Omskiy Rayon,] Irtysh, 40 km N of Omsk, Krasnoyarka, 15 September 1959, *P.I.Dorofeev s.n.* (MW); “Ad fl. Irtysch circa urb. Omsk”, 28 July 1886, *C.Golde s.n.* (LE01249647, left plant). **KAZAKHSTAN**. **Pavlodar oblysy:** “Pavlodarskiy okrug”, 1930, *Medvedev & Melvil s.n.* (AA002771).
***Acorus calamus* L**.
**RUSSIA. Omsk Oblast:** [Lyubinskiy Rayon,] Krasnoyarskoye, dried-out small lake under a mountain on the banks of the Irtysh, 31 July 1900, *N. Skalozubov 1384* (LE01249642, LE01249645); [Lyubinskiy Rayon, Kitayly] “village Kitayka (Vasin khutor)”, at the margin of a flood lake, 7 July 1920, *B.N. Gorodkov s.n.* (LE01249643); The swampy bank of an oxbow lake on the right bank of the Irtysh, 10 km above Omsk, opposite the pioneer camp, 10 July 1962, *V.B. Kuvaev 91-1* (LE01249646); Cherlakskiy Rayon, near Izylbash [Irtysh] village, floodplain of Irtysh River, 2–3 July 1932, *V. Burdakova s.n.* (TK006577); Novovarshavskiy Rayon, vicinities of Karaman aul, Irtysh River valley, 19 July 2008, *I.V. Bekisheva & S.P. Chibis s.n.* (MW0967115). **KAZAKHSTAN**. **Pavlodar oblysy:** [Zhelezin audany,] 6 km north of Urlyutub village, steep right bank of the Irtysh River, in the bushes of the swampy foot of the slope, 28 June 1955, *N.N. Tzvelev 696* (LE01224775); [Akkuly audany,] the basin of the Irtysh River, the banks of lakes and swamps on an island near Yamyshevo village, 18 August 1942, *N.V. Pavlov s.n.* (MW0812269); **Abai oblysy:** [Besqarağai audany,] the valley of the Irtysh River near stanitsa Dolonskaya [Delen], along the bottom of a dried-out small lake, 29 June 1914, *C. Kossinsky 1272* (LE01224779); [Besqarağai audany,] at the oxbow on the second terrace of the Irtysh near Dolon [Delen] village, 29 June 1955, *I. Olovyannikova s.n.* (MW0899975); [Zhanasemey audany,] left bank of the Irtysh at the confluence of the Kyzyl-Su River [Kyzylsu], clayey shore, 5 July 1914, *N. Schipczinsky 1321* (LE01224776, LE01224777, NS0040275); **Şyğys Qazaqstan oblysy:** [Şemonaiha audany,] neighbourhoods of Kamyshenka village, floodplain of the White Irtysh River, 17–22 June 1957, *Yu.A. L’vov & E.T. Sevasteeva s.n.* (TK006575); [Glubokoye audany,] near Predgornoye village, on the bank of the Irtysh River, 14 July 1929, *V. Evseenko s.n.* (TK006576); 2 km southeast of Ust-Kamenogorsk [Öskemen] Town, a swamp at the outlet of a spring, 7 July 1932, *A. Voronov 67* (MW0037511); [Bukhtarma, “ln paludosis prope Buchtarminsk.”,] [Jul.] 1840, *Karelin & Kiriloff 1066* (MW0812267, TK); [Kurchim audany,] Narymsky ridge, right bank of the Bukhtarma reservoir, lower reaches of the Kulanzhorga River, steppe, bushes and wet depressions, 414 m above sea level, 6 July 2022, *A.I. Shmakov* et al. *704* (ALTB1100061070, ALTB1100061117, ALTB1100062074); Kurchim audany, irrigation ditch in Barak-Batyra, 9 September 2019, *S.V. Smirnov & G. Bolbotov s.n.* (MW0900900, MW0899974, MW0899973); [Kurchim audany], Zaysan depression, Kurchim village, irrigation ditches, 9 September 2019, *S.V. Smirnov & G. Bolbotov s.n.* (MW0900897, MW0900898); **Jetısu oblysy:** [Alaköl audany,] Lake Alakol, near the salt marsh, in the *Haloxylon* forest, 28 June 1974, *L.Y. Kurochkina s.n.* (AA002778); [Alaköl audany,] Alakol depression, floodplain of the Tentek [Tente] River, 4 km W of Uch-Aral [Usharal] village, on marshy meadows, 10 June 1959, *V.P. Goloskokov s.n.* (AA002777, LE01224782, LE01224783, MW0812268, TK); Karatal River near Taldykorgan, on the shore, 18 June 1950, *E. Stepanova s.n.* (AA002776). **Specimens of unclear origin, labels most likely mixed:** “Right bank of Irtysh, behind the camp, Omsk”, 28 Jul. 1886, *C. Golde s.n.* (LE01249648); “Ad fl. Irtysch circa urb. Omsk”, 28 July 1886, *C. Golde s.n.* (LE01249647, right plant).
***Acorus verus* (L.) Burm.f.**
**KAZAKHSTAN**. **Şyğys Qazaqstan oblysy:** [Zaysan audany,] delta of the Old Irtysh, 20 July 1950, *I. Samusev s.n.* (AA002770); [Zaysan audany,] Lake Zaysan, Topolevsky Bay, along the edge of the floating mat in the western corner of the bay, 27 August 1943, *S.S. Pechnikova s.n.* (AA002779); [Zaysan audany,] Lake Zaysan, Topolevsky Bay, floating mat in the western corner of the lake [apparently “lake” should be read as “bay” here], 27 August 1943, *S.S. Pechnikova s.n.* (AA002780).

**Table 2:** Data on leaf anatomy, pollen stainability, seeds, nuclear DNA content and nrITS ribotypes of examined specimens. *N*cell is the mean cell number per septum in leaf lamina aerenchyma as seen in cross-section (corner cells not counted), based on counts of at least 20 septa per specimen. Legend to the field “Seeds”: A, inflorescence at anthesis; PANS, no seeds in postanthetic inflorescence; SM = I, seed morphotype I; SM = II, seed morphotype II; v, vegetative sample.

Latitude	Longitude	Specimen	Herbarium Acronym and Barcode	*N*_cell_	Pollen: % Stained (Total Counted)	Seeds	2C, pg	nrITS Ribotype and GenBank Accession No.
***Acorus americanus***								
55.5789	73.4995	*Drovorub s.n.*	LE01249644		100 (100)	A		
55.3360	73.0930	*Dorofeev s.n.*	MW			SM = I		I, PP928959
55.0468	73.1777	*Golde s.n.* (left plant)	LE01249647			SM = I		
		*Medvedev & Melvil s.n.*	AA002771	4.59		SM = I		I, PP928958
***Acorus calamus* s.str.**								
55.2216	72.9357	*Skalozubov 1384*	LE01249642	2.15	0 (105)	PANS		
55.2216	72.9357	*Skalozubov 1384*	LE01249645	2.55	0 (100)	PANS		
55.2165	73.0724	*Gorodkov s.n.*	LE01249643	2.80	1 (102)	A		
54.8309	73.3494	*Kuvaev 91-1*	LE01249646	2.70	2.8 (107)	A		
54.4922	74.3707	*Burdakova s.n.*	TK006577	2.83	17.0 (112)	A		I + II, PP928962, PP928963
54.2827	74.6188	https://www.inaturalist.org/observations/145662939 * (A.N. Efremov)				PANS		
54.2530	74.6335	https://www.inaturalist.org/observations/145662886 * (A.N. Efremov)				PANS		
54.2059	74.6756	https://www.inaturalist.org/observations/145937870 * (A.N. Efremov)				PANS		
53.8581	75.0070	*Bekisheva & Chibis s.n.*	MW0967115	2.40	0 (113)	A		I + II, PP928964, PP928965
53.7833	75.0177	*Tzvelev 696*	LE01224775			A		I + II, PP928960, PP928961
51.8863	77.3636	*Pavlov s.n.*	MW0812269	3.05		PANS		
50.6715	79.3223	*Kossinsky 1272*	LE01224779		0 (115)	A		
50.6715	79.3223	*Olovyannikova s.n.*	MW0899975	2.39	0 (129)	A		
50.3753	80.4956	https://www.inaturalist.org/observations/30665266 * (R.N. Nurkhanov)				PANS		
50.3708	80.4923	https://www.inaturalist.org/observations/30592362 * (R.N. Nurkhanov)				PANS		
50.3700	80.4919	https://www.inaturalist.org/observations/28978625 * (R.N. Nurkhanov)				PANS		
50.2975	81.3106	*Schipczinsky 1321*	LE01224776		0 (74)	A		
50.2821	81.6969	*L’vov & Sevasteeva s.n.*	TK006575		4.0 (101)	PANS		I + II, PP928966, PP928967
50.2364	82.2362	*Evseenko s.n.*	TK006576		0.9 (107)	A		
49.9385	82.6630	*Voronov 67*	MW0037511	3.60	3.0 (100)	A		
49.6030	83.5402	*Karelin & Kiriloff 1066* (left plant)	MW0812267	3.00	1.8 (113)	A		
49.6030	83.5402	*Karelin & Kiriloff 1066* (right plant)	MW812267		1.9 (104)	A		
48.9924	83.9467	*Shmakov* et al. *704*	ALTB1100061070	3.26		A	1.255	
48.9924	83.9467	*Shmakov* et al. *704*	ALTB1100062074			A	1.256	
48.5776	83.7938	*Smirnov & Bolbotov s.n.*	MW0900900		6.3 (80)	PANS	1.243	
48.5776	83.7938	*Smirnov & Bolbotov s.n.*	MW0899974	2.46	1.8 (56)	PANS		
48.5776	83.7938	*Smirnov & Bolbotov s.n.*	MW0899973	2.36	1.1 (90)	PANS		
48.5684	83.6911	*Smirnov & Bolbotov s.n.*	MW0900897			PANS	1.261	
46.4000	81.3000	*Kurochkina s.n.*	AA002778	2.68	7.0 (115)	A		
46.1744	80.8748	*Goloskokov s.n.*	MW0812268	2.25	0 (47)	A		I + II, OR542602, OR542603
44.9160	78.2460	*Stepanova s.n.*	AA002776	2.03	2.0 (101)	A		
		*Golde s.n.*	LE01249648		0.9 (110)	A		
		*Golde s.n.* (right plant)	LE01249647	2.95		V		
***Acorus verus***								
47.8625	84.5911	*Samusev s.n.*	AA002770	1.54		SM = II		II, PP928969
47.8160	84.1050	*Pechnikova s.n.*	AA002779	1.85		SM = II		II, PP928968
47.8160	84.1050	*Pechnikova s.n.*	AA002780	1.89		SM = II		

* All these URLs were accessed on 23 May 2024.

## Data Availability

High-quality images of the original material of Acorus are available through the data portal of the Herbarium of the Komarov Botanical Institute (https://en.herbariumle.ru/?t=occ, accessed on 17 June 2024) and the Herbarium of Moscow State Univeristy (https://plant.depo.msu.ru/open/public/search?collection=MW, accessed on 17 June 2024).
